# A Review on Modifications of Amniotic Membrane for Biomedical Applications

**DOI:** 10.3389/fbioe.2020.606982

**Published:** 2021-01-13

**Authors:** Fatemeh Dadkhah Tehrani, Arezoo Firouzeh, Iman Shabani, Azadeh Shabani

**Affiliations:** ^1^Cell Engineering and Bio-microsystems Laboratory, Biomedical Engineering Faculty, Amirkabir University of Technology, Tehran, Iran; ^2^Preventative Gynecology Research Center, Department of Gynecology and Obstetrics, School of Medicine, Shahid Beheshti University of Medical Sciences, Tehran, Iran

**Keywords:** amniotic membrane, composites, hydrogel, tissue engineering, regenerative medicine

## Abstract

The amniotic membrane (AM) is the innermost layer of the fetal placenta, which surrounds and protects the fetus. Its unique structure, in addition to its physical and biological properties, makes it a useful substance in many applications related to regenerative medicine. The use of this fantastic substance with a century-old history has produced remarkable results *in vivo*, *in vitro*, and even in clinical studies. While the intact or preserved AM is widely used for these purposes, the addition of further modifications to AM can be considered as a relatively new subject in its applications. These modifications are applied to improve AM properties, ease of handling, and durability. Here, we will discuss the cases in which AM has undergone additional modifications besides the required processes for sterilization and preservation. In this article, we have categorized these modifications and discussed their applications and results.

## Graphical Abstract

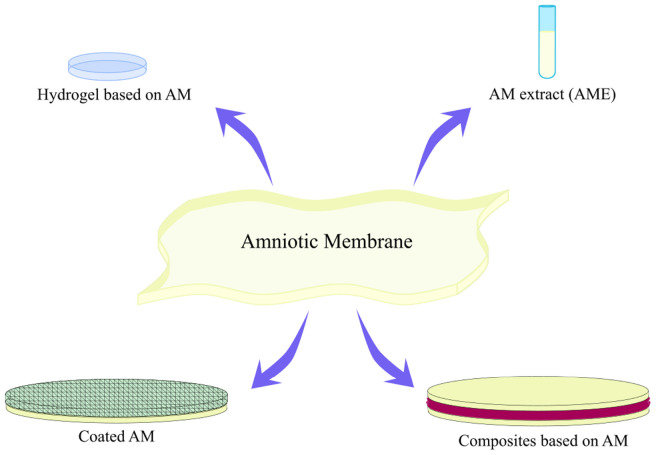

## Introduction

In the modern world, there are many incidents such as trauma or congenital or genetic disorders that can induce tissue damage or dysfunction. The body’s primary response to injury is to repair itself and maintain homeostasis for survival. This process, which is also known as tissue recovery, involves inflammation, cell proliferation, and tissue regeneration, which are all affected by cells and their microenvironment (Norouzi et al., 2015a). When an extensive injury has occurred, or functional recovery is not achieved, medical intervention is inevitable (Lewandrowski et al., 2002). One approach in these medical interventions is to replace the damaged tissue with an acceptable substitute. Depending on the type of the tissue and injury, this replacement could have a natural background such as autografts, allografts, and xenografts or could be a synthetic structure like a permanent implant. However, each of these structures is accompanied by its challenges. Tissue engineering (TE) is an alternative and promising strategy that can eliminate these limitations. Basically, in TE, cells are seeded on a scaffold, which provides temporal 3-dimensional (3D) support for cellular content and regulates their growth (Norouzi et al., 2015b). Over time, this scaffold is degraded and replaced by natural tissue. However, the utilization of any biological substitute in order to maintain, enhance, or restore tissue function is also under the scope of TE (Sharma et al., 2019). Various natural structures have the required therapeutic potential to be used as a tissue-engineered structure. Among them are the inner body membranes. Membranes actually consist of thin layers of cells or tissues that envelope the body, its internal organs, and cavities (Inci et al., 2020). Amniotic membrane (AM), Mesentery, omentum, pericardium, peritoneum, and pleura are all examples of these membranes with therapeutic applications (Inci et al., 2020). AM which is the innermost layer of the fetal membrane is a useful material with many applications in different fields of TE and regenerative medicine (Lacorzana, 2020). The application of AM has been reviewed in many articles. The most recent published reviews have focused on AM application in ophthalmology (Ehredt et al., 2019; Lacorzana, 2020), bone-related surgeries (Horn et al., 2019; Puyana et al., 2019), skin burn (Hossain et al., 2020), and skin graft (Liu et al., 2018; Liang et al., 2019). In all of these articles, the application of unmodified AM has been studied. However, as its mechanical or biological properties were not efficient enough for some studies, some authors have employed different methods to enhance its features. For instance, attaching an electrospun layer on AM (Fard et al., 2018) or even electrospinning on AM (Mandal et al., 2017; Gholipourmalekabadi et al., 2018a,b; Arasteh et al., 2020), coating an additional layer on AM (Singh et al., 2008; Murphy et al., 2019), or utilization of AM extract (AME) in the form of hydrogel (Tseng, 2016) or eye drops (Mamede and Botelho, 2015) are all considered as a modification on AM. In this article, we have reviewed different kinds of AM modifications, both in intact and decellularized form (dAM) and their applications in the medical field. In the following sections, we have first introduced AM structure and function and, after that, reviewed composite structures based on AM, AME, and hydrogels based on AM.

## Amniotic Membrane Structure, Features, and Applications

**FIGURE 1:**
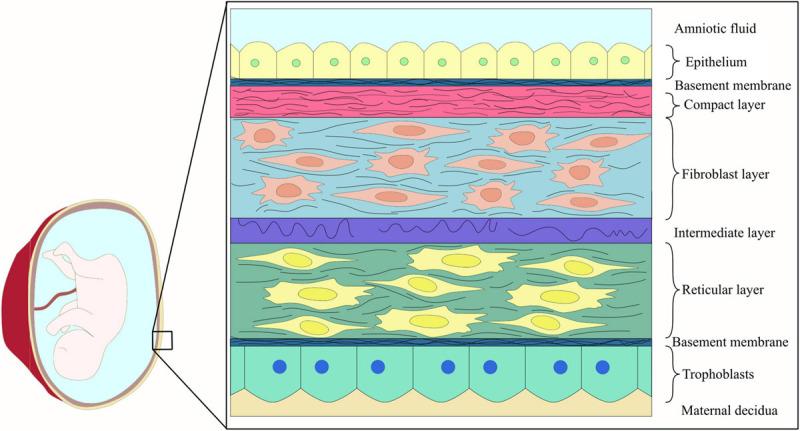
Schematic representation of fetal membrane structure.

The chorioamniotic membrane is a thin layer wrapping the developing fetus and forming the amniotic cavity (Aziz et al., 2017). It consists of two layers: chorion and amnion. The outer layer (chorion) is in contact with the mother’s cells and separated from the inner layer (amnion) by a jelly-like matrix (Mamede et al., 2012; Islam et al., 2015). AM is composed of an epithelial layer, basement membrane, and three layers of stroma (a compact, a fibroblast, and a sponge layer) (Mamede et al., 2012; Figure 1). Although the primary function of this thin, transparent, resistant, and avascular membrane is to protect the fetus from unwanted substances, bacterial infection, and trauma during pregnancy (Mamede et al., 2012; Castellanos et al., 2017), it is not just a simple barrier. One of its other functions is to transport water and soluble substances to the fetus and provide growth factors and essential cytokines for it (Mamede et al., 2012; Islam et al., 2015).

**FIGURE 2:**
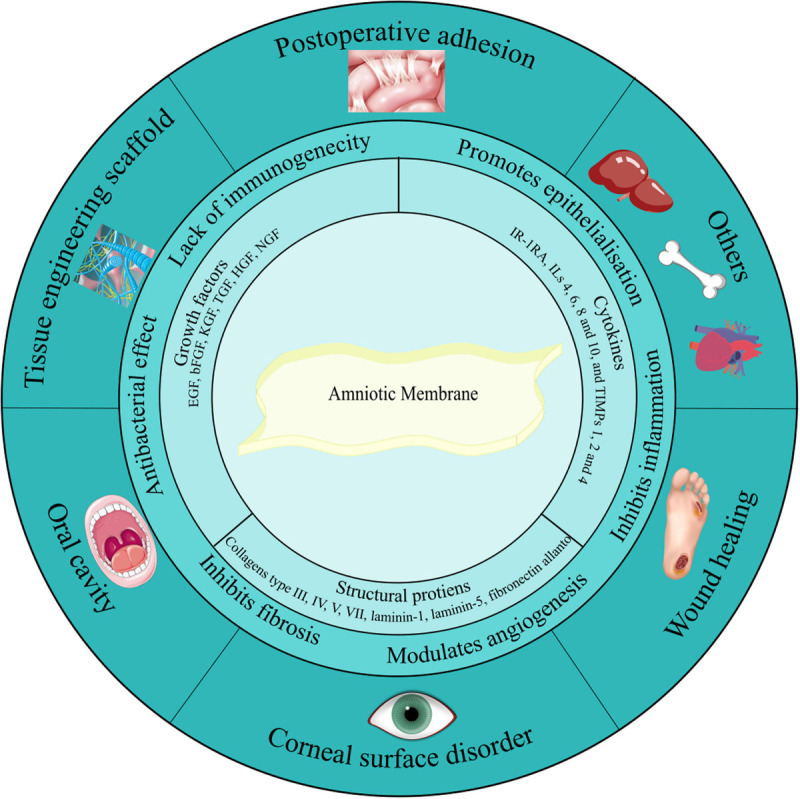
Amniotic membrane components, characteristics, and applications. Amniotic membrane is rich in growth factors, cytokines, and structural proteins. These biological factors have given AM its unique features such as antibacterial effect, anti-inflammatory activity, anti-scarring, and anti-fibrosis potential. Due to these suitable features, nowadays AM is widely utilized as a therapeutic option in the treatment of various diseases related to the urinary tract, oral cavity, skin, stomach, larynx, head and neck, ocular surface, pelvic, and abdominal surgery. Additionally, it has been used as a natural scaffold in TE.

The human AM has been widely used in TE and regenerative medicine not only due to its favorable biological and mechanical properties but also as its usage has low ethical problems (Mamede et al., 2012; Razzaghi et al., 2020). The first introduction of AM to the medical world was attributed to its similarity to human skin when Davies introduced the use of the AM in 1910 as a skin transplant. Following that in 1913, Stern and Sabella utilized AM for the treatment of skin burns and superficial wounds (Islam et al., 2015). Since then, AM has been employed for numerous applications, including disorders associated with the urinary tract (Barski et al., 2017; Fénelon et al., 2018), oral cavity (Anisuzzaman et al., 2018; Farhadihosseinabadi et al., 2018), skin (Orman et al., 2018), stomach (Iravani et al., 2020), larynx (Turchan et al., 2018), head and neck (Şapte et al., 2017; Jorge et al., 2018), ocular surface (Gheorghe et al., 2016; Nassif et al., 2016), pelvic and abdominal surgery (Lau et al., 2020), and artificial vaginal reconstruction (Mao et al., 2016; Figure 2). All of these applications are the result of the interesting features of AM (Table 1). For instance, its antimicrobial characteristic has made it a suitable option for postsurgery applications in wound healing (Tandel et al., 2018), burn injuries (Mohan et al., 2017), dental injuries (Tabatabaei et al., 2017), and ophthalmology (King et al., 2007) as bacterial infection and biofilm growth are common in these sites (Attinger and Wolcott, 2012; Chen et al., 2012; Mamede et al., 2012). On the other hand, AM’s mechanical properties are a subject of controversy. In some studies, its mechanical features are desirable, while in others it should be modified. This disagreement can be explained with the inherited variability in AM properties, which is necessary for its function, as well as the influence of preparatory methods on AM features (Chuck et al., 2004; Litwiniuk et al., 2018).

**TABLE 1:** Intact amniotic membrane biological and physical characteristics.

**Amniotic membrane features**	**Contributing factors**	**References**
**Biological properties**		
• Anti-inflammatory effect	Trapping inflammatory cells and driving them to apoptosis through its pro-apoptotic agents; production of anti-inflammatory factors by its epithelial cells; suppression the pro-inflammatory cytokines such as interleukin 1 alpha and 1 beta; production of MMP’s inhibitors; expression of migration inhibitory factor (MIF); expression of anti-inflammatory cytokines such as IL-1 receptor antagonist; secretion of anti-inflammatory factors such as PGE2, TGF-β, HGF, TNF-α, and MIF from mesenchymal and epithelial cells of AM	Mamede et al., 2012; Islam et al., 2015; Rocha and Baptista, 2015; Ilic et al., 2016
• Antibacterial and antiviral effect	Expression of natural antibacterial molecules such as β-defensins, elafin, and cystatin E; adhesion to wound surface and to act as a barrier against bacterial infiltration	Mamede et al., 2012; Islam et al., 2015; Castellanos et al., 2017; Milan et al., 2020
• Low antigenicity and non-immunogenicity	Lack of human leukocyte antigens A, B, C, and DR antigens, or β2-microglobulin on the surface of AM epithelial cells; absence of vessels, lymph, and nerves, in its structure	Mamede et al., 2012; Chopra and Thomas, 2013; Castellanos et al., 2017
• Anti-scarring and anti-adhesive effect in wound healing	Reduction of proteases activity due to the secretion of tissue inhibitors of metalloproteinases (TIMPs); the decreased activity of fibroblasts through downregulation of TGF-β with AM hyaluronic acid content	Mamede et al., 2012; Castellanos et al., 2017
• Angiogenesis and anti-angiogenesis properties (surface dependent)	Angiogenesis properties: secretion of VEGF, IL-8, angiogenin, interferon-γ, IL-6, bFGF, EGF, and PDGF by AM mesenchymal cells Anti-angiogenesis properties: secretion of IL-1, IL-2 receptor antagonist, IL-10, endostatin, TIMP-1, -2, -3, and -4, thrombospondin, and heparin sulfate proteoglycan by AM epithelial cells	Islam et al., 2015; Ilic et al., 2016
• An anticancer agent with low tumorigenicity	Secretion of pro-apoptotic agents; secretion of IL-1, IL-2 receptor antagonist, IL-10, and endostatin which all inhibit tumor growth	Islam et al., 2015; Jafari et al., 2020
• Promotion of epithelialization	Secretion of growth factors such as EGF, KGF, and HGF	Mamede et al., 2012; Islam et al., 2015; Ilic et al., 2016; Castellanos et al., 2017
• Pain reliever		Mamede et al., 2012
• Support cell adhesion and growth	Its hyaluronic acid content and proteins such as fibronectin, laminin, collagens, and proteoglycans act as a ligand for integrin receptors	Ilic et al., 2016
**Mechanical properties (intact AM)**		
• Direct tensile mechanical properties	Placental: Force before rupture: 1.2 ± 0.2 N Strain at break: 19% ± 3% N Peripheral: Force before rupture: 0.68 ± 0.08 N Strain at break: 16% ± 1% N	Litwiniuk et al., 2017
• Young’s modulus	2.29–3.6 MPa	Niknejad et al., 2008
• Tensile strength	5.475 ± 0.135 MPa	Cai et al., 2015; Ramesh et al., 2017
• Elastic modulus	4.048 ± 1.702 MPa	

The type of delivery and the region from which the AM has been extracted are the two main factors affecting AM properties (Gremare et al., 2019). Although AM contains biologically active factors such as EGF, TGF-β, and TIMP-1, in addition to structural proteins, their distribution and concentration differ from one area to another. For instance, it has been proven that to ease fetal membrane rupture through delivery, AM has a weak zone overlying the cervix (Litwiniuk et al., 2017). Overall, in the case of physiological delivery-derived AMs, the placental portion has a more stimulatory influence on fibroblast and keratinocyte cell lines as it is rich in EGF and TGF-β; hence, it supports chronic wound healing (Brown et al., 1989; Ogawa et al., 2004; Gicquel et al., 2009). On the other hand, the cervical area of AM assessed from cesarean delivery, which has a low level of TGF-β, is more suitable for ophthalmologic applications (Brown et al., 1989; Kane et al., 1991). TGF-β is highly essential during all phases of wound healing, but its overexpression, especially during eye surgery, can cause fibrosis and hypertrophic scar formation, which in the case of corneal damage may lead to a subsequent loss of corneal transparency (Branton and Kopp, 1999; Papakonstantinou et al., 2003; Fairbairn et al., 2014; Torricelli et al., 2016). In the case of mechanical properties, according to a recent study, placental AM is much stronger and stretchable than peripheral AM by an average of 82 and 19%, respectively.

Aside from delivery mode, AM processing has a direct effect on its features. AM properties can be tailored only by changing the processing method (Aziz et al., 2017). In general, for AM clinical applications or its preservation in tissue banks, it is crucial to perform donor screening and selection, procure the membrane, wash it, and perform additional processing steps. It is common to treat the AM chemically or with antibiotic substrates, preserve, sterilize, package, and store it (Mamede et al., 2012). The reason for epithelial layer removal, AM sterilization, and its preservation are, respectively, to reduce graft rejection, minimize the risk of disease transmission, and store it more quickly for a more extended period (Gholipourmalekabadi et al., 2019a; Khosravimelal et al., 2020). Although AM-derived cells do not express immunogenic molecules, it has been reported that fresh AM can cause some inflammatory responses (Gholipourmalekabadi et al., 2019a); thus, it may be a better choice for some experiments to remove the cellular content of AM. There are different agents for AM decellularization, such as sodium dodecyl sulfate (SDS), urea, EDTA, and trypsin (Ilic et al., 2016). AM can be stored in a fresh, cryopreserved, or dried form. It can become cryopreserved with glycerol or DMSO and dried by freeze-drying, air, or oven-drying (Mamede et al., 2012); however, studies have shown that biological, morphological, and physical properties remain more intact with freeze-drying and storage in glycerol (Lacorzana, 2020).

Although all of the mentioned factors influence AM features, it is safe to say that AM in all forms (intact, dried, decellularized, and frozen) has unique properties suitable for many applications (Liu et al., 2018; Ehredt et al., 2019; Horn et al., 2019; Liang et al., 2019; Puyana et al., 2019; Hossain et al., 2020). Moreover, in some cases AM has been added to another material to improve its biological characteristics (Jiang et al., 2007). However, its mechanical and sometimes biological properties are sometimes not sufficient. To enhance these features, it is possible to apply AM in combination with another material. In the following sections, we have reviewed these modifications and categorized them into three main groups: composites based on AM, AM extract (AME), and hydrogels based on AM. The information given in each part is summarized in a table following that section.

## Composites Based on Amniotic Membrane

As it was mentioned previously, AM’s ECM is rich in structural proteins with a variety of biochemical cues and has been successfully utilized as a basement membrane substitute (Sekiyama et al., 2007). However, its inherent limitations, such as poor mechanical properties, short-term therapeutic efficiency, the difficulty of handling and suturing during surgeries, and inefficient adhesive properties, highlight the need for modifications (Cai et al., 2015). To overcome the limitations associated with AM, some authors considered the development of biocomposites based on AM by the addition of polymers, fibrin glue, or any other material to it. On the other hand, sometimes AM is utilized in composite form just to enhance the biological properties of another material. In this section, we have categorized these composites in three subgroups: coated AM, AM as a coating, and composites based on pulverized AM. Following this, we will discuss the advances in this field and summaries them in Table 2.

**TABLE 2:** Summary of composites based on AM.

**Author, year**	**Therapeutic goal**	**Experimental settings (target tissue/cells)**	**Secondary biomaterial**	**Conclusion**	**References**
Uchino, 2006	Artificial cornea scaffold	*In vitro* (rabbit corneal epithelium)	PVA	PVA-AM is a biocompatible hybrid material for keratoprosthesis	Uchino et al., 2007
Jiang, 2007	Intravascular stent	_	SS stent	AM is an excellent elastic material for stent covering and has a good blood compatibility	Jiang et al., 2007
Sekiyama, 2007	Ocular surface reconstruction	*In vivo* (rabbit)	FG	FG-coated AM retains most of the biological characteristics of freeze-dried AM and is a safe, simple, and useful transplant for ocular surface reconstruction	Sekiyama et al., 2007
Singh, 2008	Burn dressing	_	Silver	Deposition of silver particles on AM results in the formation of an antibacterial barrier with controlled release of moisture vapor and a high absorption capacity	Murphy et al., 2019
Washburn, 2010	Abdominal adhesion prevention	*In vivo* (Sprague Dawley rat)	Halofuginone and chitosan	AM coated with halofuginone alone or in combination with chitosan resulted in lower adhesion rate	Washburn et al., 2010
Adamowicz, 2015	Reconstructive urology	*In vitro* and *in vivo* (MSC and Wistar rats)	PLCL	Frozen AM sandwiched between two layers of electrospun PLCL can support urothelial cells and SMC regeneration and is suitable for reconstruction of the urinary bladder wall	Adamowicz et al., 2016
Cai, 2015	Ocular surface reconstruction	*In vivo* (rabbit)	FG	FG-double-layered AMT has excellent stability and short operating time and promotes a stable and rapid reconstruction of the ocular surface	Cai et al., 2015
Hortensius, 2016	Tendon regeneration	*In vitro* (equine tenocytes)	CG	Incorporation of dAM into CG-based scaffold results in a modified inflammatory response of the target tissue	Hortensius et al., 2016
Najibpour, 2016	Abdominal hernias	*In vivo* (Dutch white rabbits)	PP mesh	Addition of AM to PP mesh results in less adhesion and inflammation, higher epithelialization, and wound healing improvement	Najibpour et al., 2016
Mandal, 2017	Ocular surface	*In vitro* (3T3 and (HEK)-293)	Clavanin A	A-coated dAM reduces biofilm formation while has no significant cytotoxicity	Singh et al., 2008
Becker, 2018	Cardiac TE	*In vitro* (human cardiac fibroblasts, epicardial progenitor cells, murine HL- cells, and human immune cells)	hcECM	Cell adhesion, proliferation, and viability of dAM increased after it was coated with hcECM and less inflammatory response was observed	Becker et al., 2018
Hortensius, 2018	Tendon regeneration	*In vitro* (MSC)	Collagen scaffold	The addition of dAM to collagen-based scaffolds as bulk incorporation or a membrane wrap results in a biomaterial with both a tendon-mimicking structure and an immunomodulatory effect	Hortensius et al., 2018
Liu, 2018	LSC deficiency	*In vitro* (primary rabbit LSCs and bone-mouse marrow-derived macrophages)	Polymeric fiber mesh	The composite membrane based on lyophilized dAM and nanofiber mesh offers superior mechanical features as well as necessary biochemical cues for LSC attachment, growth, and maintenance	Fard et al., 2018
Rashid, 2018	Abdominal wall hernias	*In vivo* (Wistar albino rats)	PEG+PP mesh	Coverage of PP mesh with BAM and 5% PEG results in the lowest adhesion percentage	Rashid et al., 2018
Soylu, 2018	Abdominal wall defect	*In vivo* (Wistar albino rats)	PP mesh	Addition of AM to PP mesh results in less intra-abdominal adhesions, less inflammation, and higher epithelialization	Soylu et al., 2018
Aslani, 2019	Vascular tissue engineering	*In vitro* (HUVEC and MSC)	PLLA-ASA	AM-coated ASA-loaded aligned electrospun scaffold supports endothelial differentiation and provides superior biocompatibility with appropriate signals needed by EC	Aslani et al., 2019
Gholipourmalekabadi, 2019	Modulation of hypertrophic scar formation	*In vitro* and *in vivo* (human ADSCs, rabbit ear model)	Silk fibroin	AM/silk minimizes the post-injury hypertrophic scar formation through decreasing the collagen deposition and increasing MMP1 expression and deposition	Gholipourmalekabadi et al., 2019b
Ramakrishnan, 2019	Wound healing	*In vitro* (dermal fibroblasts)	PLGC+PEG+ SNP+fibrin	Combination of AM-F-PLGC-SNP can be advantageous not only for wound coverage but also for skin tissue regeneration	Ramakrishnan et al., 2019
Zhang, 2019	Oral defects	*In vitro* and *in vivo* (human fibroblasts, CAM assay, New Zealand white rabbits)	GelMA	Composition of GelMA and particulated AM resulted in an easy to synthesize, store, and handle substrate suitable for the treatment of oral mucosal defects	Zhang et al., 2019
Zhou, 2019	Corneal epithelial defect	*In vivo* (rabbit)	PCL	PCL-dAM composite has pro-regenerative and immunomodulatory properties of dAM and with a lower degeneration rate	Zhou et al., 2019
Adamowicz, 2020	TE of the urinary bladder	*In vitro* (SMC derived from porcine detrusor and porcine UC)	Graphene layers	Intact AM covered with solid graphene layers has the potential to obtain electrical stimulation for smooth muscle layer	Adamowicz et al., 2020
Akyürek, 2020	Prevent capsule contraction in Silicone breast implants	*In vivo* (Wistar rats)	Silicon	Coating silicone implants with AM reduces capsule thickness in comparison with bare silicon	Akyürek et al., 2020
Dewey, 2020	Bone repair	*In vitro* (pASC)	Collagen scaffold	Collagen-dAM composite scaffold is potentially suitable for craniomaxillofacial bone repair especially in the presence of inflammation	Dewey et al., 2020
Yang, 2020	Wound healing	*In vitro* and *in vivo* (human foreskin fibroblast cells and mice)	Chitosan	Double-layer membrane based on dBAM and chitosan is a biocompatible structure with potential benefits in healing full-thickness diabetic patients	Yang et al., 2020

### Coated Amniotic Membrane

The unique properties of the AM have encouraged researchers to use it for a variety of applications. In some cases, other biological or non-biological components are coated on the AM to improve its properties and performance. The secondary agent can be in the form of particles, gels or biological glues, and a polymeric or an electrospun layer. In some studies, the secondary agent has been only placed on the AM surface, while in others, it has been immobilized on the surface of AM through a chemical reaction.

#### Amniotic Membrane Coated With Particles

Biological or chemical particles can be coated on the membrane structure through various processes such as peptide self-assembly (Singh et al., 2008) and deposition (Murphy et al., 2019). These modified constructs have mainly been utilized to improve the antimicrobial effect of AM (Singh et al., 2008; Murphy et al., 2019). For instance, in one study, Mandel et al. coated dAM with clavanin A, after it was modified with self-assembly, to prevent biofilm formation over the membrane. Based on their study, the A-coated dAM had better biocompatibility as well as lower cell attachment colonization and fungal colonization (*p* < 0.05) in comparison with the dAM. Moreover, it showed excellent physical, morphological, and antifungal characteristics, which made it suitable for ocular surface infection control (Singh et al., 2008). In another study, Singh et al. improved the antibacterial property of AM by silver deposition. *In situ* reduction was the method of choice for this deposition and resulted in the formation of a barrier against the penetration of bacteria. Additionally, this structure obtained a controlled release of moisture vapor and showed a great absorption capacity (Murphy et al., 2019).

#### Amniotic Membrane Coated With Gels and Biological Glues

Another approach to ameliorate AM properties in a composite form is to combine it with a gel or a biological glue. These composites are mainly designed to improve the adhesive and biological properties of AM. For instance, AM has been extensively utilized for ocular surface reinstruction; however, due to inefficient mechanical properties of fresh and dried AM, single-layer transplantation of AM has proven to be insufficient. Moreover, merely adding multiple layers of AM is not enough as they require excessive stitches to remain attached to each other and the surface of the eye (Brücher et al., 2020). However, by adding fibrin glue (FG) to this structure, it is possible to enhance the mechanical properties of AM (tensile strength: 0.727 MPa, strain at break: 24.130%) while reducing the need for stitches. Moreover, FG-double layer AM improves the rate of epithelial healing (Cai et al., 2015). Fibrin glue has also been added to AM just to avoid sutures for binding AM to the ocular surface (Sekiyama et al., 2007). In another experiment, the effect of chitosan and/or halofuginone gel coating on AM to reduce tissue adhesion was studied by Washburn et al. (2010). They evaluated this effect on a rat uterine injury model and concluded that AM both as a single layer or coated with gel reduced moderate and severe tissue adhesion. Likewise, when AM was coated with halofuginone alone or in combination with chitosan, the percentage of adhesions declined.

To enhance the biological properties of AM, a novel composite patch based on dAM has been developed for cardiac TE (Becker et al., 2018). This structure was prepared by combining a hydrogel obtained from human cardiac ECM (hcECM) with dAM via the dry-coating procedure. According to the results of this study, coating dAM with hcECM modified the regenerative properties of the dAM so that it would be better suited for applications related to the heart. This composition did not alter the mechanical properties of the dAM, as they can affect cell behavior. Moreover, the tissue-specific protein composition of the myocardial ECM, which is essential for its biological activity as a lineage supporter and cytoprotector, remained intact. Overall, based on their results, this scaffold may be a potential platform for the epicardial delivery of cells and therapeutic agents as it has superior adhesion capacity, supports cell proliferation and viability, and modulates inflammatory responses.

#### Amniotic Membrane Coated With a Layer

**FIGURE 3:**
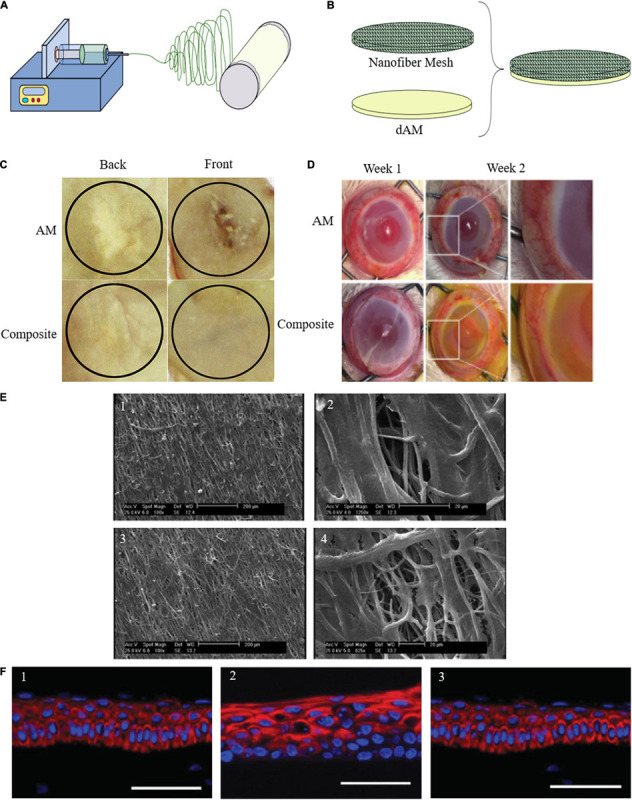
**(A)** Schematic representation of direct electrospinning of secondary material on AM. **(B)** Conjugation of the surface-activated nanofiber mesh on the AM. **(C)** Gross healed wound areas after 30 days of wound healing from front and back views comparing AM and composite effect [reproduced with permission from Mandal et al., 2017. Copyright 2020 Elsevier]. **(D)** Maintenance of structural integrity, reduction of vascularization, and degradation after PCL-dAM composite transplantation in comparison with the AM-treated group for treating alkali-burn induced LSCD model [reproduced with permission from Zhou et al., 2019. Copyright 2019 Elsevier]. **(E)** Attachment and infiltration of Wharton’s jelly-derived MSCs seeded on the PLLA scaffolds after 7 days in two different magnification. (1, 2) Aligned PLLA scaffold containing ASA. (3, 4) Aligned PLLA scaffold containing ASA and coated with AM lysate [reproduced with permission from Aslani et al., 2019. Copyright 2019 Wiley]. **(F)** Representative series of expression of corneal epithelium-specific keratin 3 (K3) in epithelial cells cultured on (1) PVA-AM, (2) PVA-collagen, and (3) normal rabbit cornea [reproduced with permission from Uchino et al., 2007. Copyright 2007 Wiley].

The most widely used approach for enhancing AM properties is to utilize multilayered constructs of AM in combination with a polymeric layer, which can be constructed in a variety of ways. One strategy is to combine AM with an electrospun layer either by direct electrospinning the secondary material on the AM or conjugating the surface-activated nanofiber mesh on the AM (Figures 3A,B). Another method is to add a casted layer on top of AM.

Silk is one of the natural biomaterials that have been added to AM to improve its mechanical properties as well as degradation rate (Gholipourmalekabadi et al., 2018b). Silks’ biocompatibility, non-cytotoxicity, low immunogenicity, ease of manipulation, low rate of biodegradability, and mechanical and structural superiority have paved the way for its extensive application in biomedical applications and wound healing (Mandal et al., 2017; Gholipourmalekabadi et al., 2018a,b, 2019b). Thus, in some cases, especially for skin regeneration, silk nanofibers were electrospun on dAM (Figure 3C; Mandal et al., 2017; Gholipourmalekabadi et al., 2018a,b; Arasteh et al., 2020). In addition to better maintenance of the 3D structure, these constructs supported cell adhesion (Gholipourmalekabadi et al., 2018b), keratinocyte differentiation (Gholipourmalekabadi et al., 2018a), and skin regeneration. Moreover, while they regulated inflammation (Mandal et al., 2017), no detectable cytotoxicity had been observed (Gholipourmalekabadi et al., 2018b). According to Gholipourmalekabadi et al. (2019b) AM/silk had the same effect on cell viability and cytotoxicity as simple AM *in vitro*. Additionally, they showed that AM/silk minimized the post-injury hypertrophic scar formation in the rabbit ear model *in vivo* as it decreased the collagen deposition while it increased MMP1 expression and deposition.

As was mentioned in the previous section, improving mechanical properties and regulating the degradation rate of AM is necessary for its practical application in the treatment of ocular surface disorders. For instance, limbal stem cell (LSC) expansion is one of the most promising areas for AM applications, yet challenges have always accompanied it due to poor mechanical characteristics of AM (Serna-Ojeda et al., 2020). To overcome this challenge, Liu et al. developed another composite structure based on AM to enhance its tensile property and toughness. They evaluated its effect on the treatment of LSC deficiency and corneal injuries (Fard et al., 2018). This structure, which was based on dAM and a fiber mesh, combined the biochemical activity of dAM necessary for LSC adhesion, growth, and maintenance with a mechanically stable structure (Liu et al., 2018; Zhou et al., 2019). In this study, three polymers (PLA, PLGA, and PCL) underwent electrospinning to form three nanofiber meshes, and following that, each of them was grafted with PAAc chains. Based on Liu et al. findings, the composite membrane was easier to manipulate in comparison with fresh and freeze-dried dAM; retained dAM support of rabbit LSC attachment, proliferation, and maintenance; and regulated inflammatory response for 7 days and had anti-inflammatory properties. Based on their results, elastic modulus, strain to failure, ultimate tensile strength, toughness, and failure force with suture of all the composite scaffolds were much higher than dAM. However, the PCL fiber-dAM scaffold possessed more balanced mechanical properties for the application in LSC transplantation (Liu et al., 2018). In another study, Zhou et al. employed PCL-dAM composite to improve LSC expansion in the rabbit corneal epithelial defect model (Figure 3D). They have reported that this composite membrane maintained the pro-regenerative and immunomodulatory properties of dAM and, at the same time, reduced its degeneration rate by 40%, which means it can provide lasting coverage in the defect site (Zhou et al., 2019).

Amniotic membrane has also been utilized in the field of urological TE due to its potential to support smooth muscle cell (SMC) regeneration and induce epithelialization (Sharifiaghdas et al., 2009; Adamowicz et al., 2012). Adamowicz et al. (2016) have developed a biocomposite material based on frozen AM and electrospun membranes for the regeneration of the bladder wall. This structure was constructed from a frozen AM sandwiched between two layers of electrospun poly(L-lactide-co-ε-caprolactone) (PLCL). According to Adamowicz et al., PLCL layers not only improved the mechanical properties of AM but also promoted the cellularization of AM by the host’s cells. Furthermore, based on their report, this biocomposite material induced the formation of a multilayered bladder wall similar to native bladder but stiffer (lower Young’s modulus by nearly two folds) within 12 to 14 weeks.

Another strategy is to add silver nanoparticles as well as an electrospun layer, to the AM to improve its antibacterial effects. For example, in one study, Ramesh et al. (2017) electrospun a combination of umbilical cord collagen and green silver nanoparticles on cross-linked dAM to construct a hybrid biological nano-scaffold with long shelf-life for wound healing. The green silver nanoparticles, which were prepared with reducing silver nitrate by Curcumin, promoted scarless healing as silver modulates inflammatory response, and curcumin is a wound-healing agent. Their *in vitro* assessment showed that this scaffold was efficient for the differentiation of human cord blood-derived stromal cells (CBMSCs) to keratinocytes and skin fibroblasts. Also, they provided evidence that this dressing enhances the wound healing process (almost 100% after 21 days – with the formation of hair follicles and sweat glands and epithelialization in some wounds) and minimizes scar formation in small animal models. According to their results, although the AM was superhydrophobic, the electrospun layer retained moisture. Additionally, this dressing, which had a superior tensile strength in comparison with native AM (7.96 ± 3.06 MPa), provided a sustained/controlled release of silver and needed fewer dressing changes (Ramesh et al., 2017).

In another study, Ramakrishnan et al. also added silver antibacterial properties to the composite structure based on AM and an electrospun layer with improved handling properties. Their proposed scaffold was prepared with electrospinning of the poly-(lactide-co-glycolide-co-caprolactone) (PLGC) terpolymer after it was incorporated with PEG-protected SNPs, on a layer of fibrin and AM (Ramakrishnan et al., 2019). This biodegradable combinatory scaffold with biological cues was used as a wound dressing for dermal regeneration. According to their results, the properties of this scaffold are superior to each component alone as it had the mechanical strength of PLGC, suitable biological properties of AM, cellular stimulatory effect of fibrin, and antimicrobial property of SNPs. The tensile strength (3.62 ± 0.4 MPa), elongation (10.6 ± 4.6 MPa), modulus (67.3 ± 19.6 MPa), and swelling percentage of composite scaffold improved in comparison with AM. On the other hand, the addition of AM supported fibroblast attachment and growth on the PLGC-SNP scaffold for 14 days.

Another example of multilayered AM composite with an electrospun layer for improved biological properties has been developed for vascular TE. Engineering small-diameter vascular graft is more challenging than large ones. While for large-diameter arteries, bio-stable and mechanically strong synthetic grafts have been developed and used successfully, small-diameter vessels require a more biocompatible vascular graft with precise structural, biophysical, and topographical design (Ravi and Chaikof, 2010; Aslani et al., 2019). To achieve this goal, Aslani et al. (2019) fabricated an electrospun poly(L-lactic acid) (PLLA) scaffold containing an anticoagulation agent (acetylsalicylic acid-ASA) and coated its surface with AM lysate prepared with the digestion of AM in an enzymatic solution made from HCl and pepsin, which is rich in basement membrane proteins and glycoproteins. The inner surface of their proposed scaffold supported endothelial differentiation, which is a natural anticoagulant. Among the fabricated scaffolds they have studied, aligned ASA-loaded AM lysate-coated scaffolds were the best option, which supported endothelial cell differentiation (Figure 3E). In this study, AM was utilized to improve the overall cytocompatibility of the scaffold for human umbilical vein endothelial cell (HUVEC) culture and endothelial differentiation of MSC.

In a new study, a wound dressing based on decellularized bovine AM (dBAM) and sponge-like chitosan membrane (BAMCSM) has been developed to accelerate diabetic wound healing (Yang et al., 2020). This biomaterial, which has been fabricated via the freeze-casting method, consisted of two layers of sponge-like chitosan and a layer of dried dBAM. The porous chitosan scaffold meliorated blood coagulation and swelling properties, while dBAM provided the essential growth factors and collagen content for wound healing. To retain the biomedical and architectural properties of dBAM, poly(ethylene glycol) diglycidyl ether (PEGDGE) was applied as a cross-linking agent. According to their data, this wound dressing had better biocompatibility, air permeability, improved swelling ability, and mechanical properties in comparison with each material alone. Additionally, this membrane promoted diabetic wound healing ratio (87.67% at day 8 on average) to the stage that even sebaceous gland, hair follicles, and collagen fibers with parallel organizations were observed (at day 14).

In addition to this, following their previous study, Adamowicz et al. (2020) introduced a new composite biomaterial based on AM and graphene to create an interface between cells and external stimuli to replace neural network for urinary-bladder TE. To evaluate the properties of this structure, they seeded it with SMCs and urothelial cells. According to their published results, the growth of SMCs increased due to the electrical stimulation applied through the biocomposite structure. Moreover, they observed *in vitro* contractile response of SMCs, which indicates the effectiveness of this structure in transferring electrical stimulation.

### Amniotic Membrane as a Coating

In some cases, AM is used as a coating to improve the biocompatibility of other materials. Currently, the conventional method for hernias treatment is the use of polypropylene (PP) mesh, which causes some complications such as tissue adhesion (Najibpour et al., 2016). In several *in vivo* models (rat and rabbit), the AM has been used as a coating on PP mesh without any suture or addition of adhesive material. The effectiveness of AM-coated PP mesh in comparison with single PP mesh on the prevention of abdominal adhesions was assessed. Findings show less adhesion and inflammation, higher epithelialization, and wound healing improvement when the AM was applied (Najibpour et al., 2016; Soylu et al., 2018). Rashid et al. conducted a similar study in the rat model. They used BAM with a coating of PEG on the abdominal side of the PP mesh. The lowest adhesion percentage was observed in the experimental group in which PP mesh was covered with BAM and 5% PEG. However, this study shows that BAM alone was not as efficient (Rashid et al., 2018).

In another study, Jiang et al. (2007) fabricate AM-covered stainless steel (SS). In this study, Jiang et al. compared AM to porcine small intestinal submucosa (PSIS), which had previously been utilized as a stent coverage (Toyota et al., 2002). Although stretch stress tests showed that cryopreserved AM is not as extensible as PSIS (AM in comparison with PSIS: tearing length of 9.39 and 12.95 mm; maximum stress of 3.99 and 12.94 MPa; maximum strain of 0.47 and 0.65 mm/mm), stress–strain curves indicated that AM is more consistent than PSIS and is an excellent elastic membrane as a cover for stent as it also is blood compatible and has minimum immune response. According to their results, internally AM-covered stent kept the arterial lumen smooth while it had a better chance of AM detachment. On the other hand, externally AM-covered stent connected the stent firmly to the vessel with minor vessel injury but it did not have any effect on enhancing arterial lumen smoothness.

In another study, AM has been utilized in a composition with polyvinyl alcohol (PVA) hydrogel to improve PVA’s biological properties. PVA is one of the well-suited candidates for corneal transplantation, but its limited biocompatibility has made some challenges for its *in vivo* application. To address this problem, Uchino et al. (2007) designed a hybrid polymer based on PVA hydrogel and dAM. In order to do this, collagen immobilized-PVA hydrogel was fabricated and coated with AM using a tissue adhesive component consisting of collagen and citric acid as a cross-linker. This structure improved corneal epithelialization after 2 weeks in comparison with PVA-collagen hybrid, which resulted in epithelium loss in the same period (Figure 3F).

The most recent study on AM as a coating was conducted by Akyürek et al. (2020) who evaluated the efficiency of AM-coated silicon breast implant on capsule formation *in vivo*. Based on Akyürek et al. hypothesis, the anti-inflammatory and anti-fibrinolytic effect of AM can be useful in the prevention of the most severe complication in silicone breast implants, which is capsule contraction. According to their results, AM retained its integrity after 3 weeks in 80% of rats, but it was not detected after 12 weeks. However, their results show that composite implants which remained for 12 and 24 weeks significantly reduced capsule thickness in comparison with bare silicon (p:0.015, p:0.012) while the difference between capsule thickness after 3 weeks was not statistically significant between two groups (p:0.674).

### Composites Based on Particulated Amniotic Membrane

Another approach that has been developed by some studies is to enhance the biological properties of other materials by the addition of particulated AM (pAM) before the final fabrication process. Different tools have been utilized for AM pulverization, which is all accessible and straightforward, such as mortar and pestle (Dewey et al., 2020), and tissue grinder (Zhang et al., 2019). One example of these constructs has been developed for the treatment of oral mucosal defects, which has always been challenging for AM. Zhang et al. (2019) have introduced an alternative approach for treating these defects using dAM in combination with methacrylated gelatin (GelMA). This composite substitute was prepared with the addition of decellularized amniotic particles (dAP) to GelMA solution following with a curing process initiated by photosensitive acylphosphinate. Their proposed structure has the mechanical strength (maximum load value of 1.04 ± 0.03 MPa) and adhesion of GelMA blended with biological cues of dAM. Their results show that this scaffold significantly increased the number of neovascularization in a chick chorioallantoic membrane (CAM) assay (more than 10 mm^2^ compared to GelMA). Additionally, while it did not cause any postoperative infection or allergy reaction in rabbit models, this 3D porous scaffold improved angiogenesis and was suitable for the treatment of oral mucosal defects.

Hortensius et al. have developed another composite structure based on pAM for tendon regeneration. In two studies, they combined collagen-glycosaminoglycan (CG) scaffolds with dAM to promote tendon repair. In the first study, they hypothesized that the addition of the ECM found in low inflammatory environments to the scaffold would modify the host immune responses (Hortensius et al., 2016). To examine this, they incorporated chondroitin sulfate (CS), hyaluronic acid (HA), and particulate dAM into collagen suspensions with different ratios and fabricated the final scaffolds with freeze-drying. According to their results, scaffolds containing HA or dAM increased the metabolic activity of tenocytes in comparison with other scaffolds, especially in high inflammatory media after 7 days. Besides, their findings show that scaffolds based on dAM and HA maintain their anti-inflammatory features within a collagen-based scaffold and alter the pro-inflammatory response associated with scar formation during tendon healing. However, these scaffolds did not have the required mechanical strength to be utilized for direct tendon regeneration (elastic modulus of 1.065 ± 0.083 KPa). In the next study, they explored two different methods for the fabrication of these scaffolds (Hortensius et al., 2018). The first one was the same as the fabrication method used in the first study, and the other method was based on the traditional collagen-chondroitin sulfate (C/CS) scaffold with a layer of dAM wrapped around it. According to their results, these scaffolds affected the response of MSCs to inflammatory challenges in the early stages, and it may be a potential biomaterial for enhancement of tendon regeneration.

Another composite structure based on pAM and mineralized collagen scaffold has been developed recently by Dewey et al. for bone repair. The fabrication method of this scaffold was similar to the one described by Hortensius et al. in terms of dAM pulverization before adding it to collagen suspension; however, in this study, the composition of the collagen scaffold was slightly different from those reported by Hortensius et al. (Dewey et al., 2020). Based on the results reported by this group, the addition of dAM to collagen scaffold resulted in smaller pore size (approximately 60 μm) and higher Young’s modulus, collapse stress, and collapse strain. Additionally, while the collagen scaffold without any dAM supported cell viability and osteogenic differentiation more, the final mineral formation and osteogenesis in response to the inflammatory challenge was enhanced in mineralized collagen-AM scaffold after 28 days.

## Amniotic Membrane Extract

Despite beneficial chemical and physical characteristics of AM, a significant problem associated with its utilization is the difficulty in providing fresh AM (Kang et al., 2013). Different approaches have been developed to increase AM shelf life, such as freeze-drying or cryopreserving the AM. An alternative approach is to use amniotic membrane extract (AME). AME contains almost all of the therapeutic components of the cryopreserved AM (Dudok et al., 2015); it is rich in growth factors such as EGF, HGF, bFGF, protease inhibitors, and HC-HAPTX3, which is a matrix component with ant-inflammatory, anti-angiogenesis, and anti-scarring effects (Mahbod et al., 2014; Mamede and Botelho, 2015; Stachon et al., 2015). Furthermore, AME can be easily preserved and be sterilized through filtration.

Additionally, as a result of a study conducted on the antibacterial effect of AME/CME (chorionic membrane extract) against *S. pneumoniae*, it has been proven that as well as AM/CM, AME/CME has several antimicrobial peptides and proteins that inhibit bacterial cell growth and biofilm formation (Yadav et al., 2017). These extracts, in combination with P–S antibiotic solution, inhibit *in vitro* biofilm growth and eradicate pre-established biofilms. This effect has been further investigated by Park et al. (2020) in a recent study. They aimed to assess the AME effect on the growth of the middle ear (ME) mucosa in response to otitis media (OM) induced by non-typeable Haemophilus influenzae (NTHi). According to their results, AME influenced mucosal proliferative response in a dose-dependent manner. However, due to the limitations of this study, it is hard to interpret their results.

The anti-inflammatory effect of AM is preserved in its extract form by the same pathway, which is the induction of macrophage apoptosis (Li et al., 2006). He et al. (2008) proved this activity by studying RAW264.7 morphological alternation, cell growth, and apoptosis in resting and activated macrophages in a medium containing AME. Their results indicate a reduction in cell spreading mediated by a reduction in actin filament intensity in AME-treated cells, suppression of cell growth, and cell apoptosis induction. According to He et al. (2009) and Shay et al. (2011), the main component which is partially associated with the anti-inflammatory and anti-scarring effect of AME is HC-HA complex (hyaluronan and heavy chains of inter-α-inhibitor). In another study, Laranjeira et al. evaluated the anti-inflammatory effect of AME on T cells and antigen-presenting cells. According to their results, AME inhibits the inflammatory response of T cells and reduces the proportion of T cells that produce cytokines (Laranjeira et al., 2018). However, this effect is not comprehensible for APCs. Overall, they showed that the anti-inflammatory effect of AME is mostly due to its direct effect on the proliferation capacity of T cells in response to mitogen activation. Additionally, it inhibits the expression of proteins that have a cytotoxic function.

### Preparation of Amniotic Membrane Extract

**FIGURE 4:**
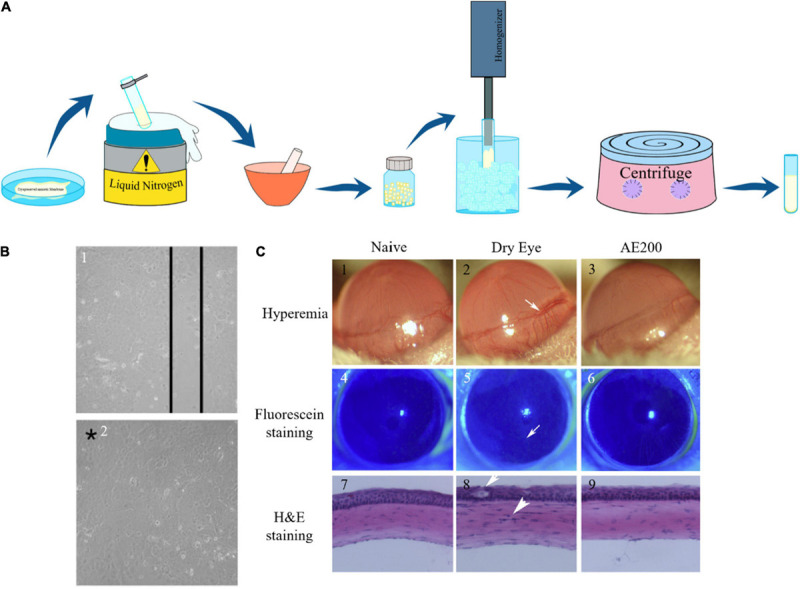
**(A)** Schematic representation of AME preparation. **(B)** Results of linear mechanical abrasion test performed on control and AME-treated HCE cells after 72 h. After 72 h of mechanical abrasion, confluency was reached in AME-treated plates showing the healing effect of AME on mechanical cellular injury. Confluence is noted by the asterisk [reproduced with permission from Dudok et al., 2015. Copyright 2015 Wiley]. **(C)** Proof of reduction of ocular surface abnormalities induced by BAC with AE. The dry eye group was left untreated, and 200 μg/ml AE was administered for the AE200-treated group 3 times daily. On the 6th day, hyperemia was evaluated, and fluorescein staining and H&E staining were also performed. The white arrow in (2) shows ciliary hyperemia in dry eye condition, that in (8) shows the epithelium was weakened, and the other one shows the infiltration cell. Corneas treated with AE had smoother epithelium and less inflammation [Reproduced with permission from Xiao et al., 2013. Copyright 2013 Elsevier].

Different approaches have been developed for the preparation of AME without consistent standardization. The most convenient method consists of washing previously isolated and screened AM with a sterile saline solution containing 1% antibiotic cocktail (penicillin, streptomycin, and neomycin), submerging AM in the liquid nitrogen, slicing the frozen AM into small pieces, and manually morselizing it to a fine powder and homogenizing it with normal saline or PBS. Following that, the mixture is centrifuged. The supernatant is collected and centrifuged again (Figure 4A) and finally sterilized by passage through a filter. In other methods, cryopreserved or dehydrated AM is micronized or pulverized (Murri et al., 2018). In some cases, AME was obtained from a decellularized AM (Shakouri-Motlagh et al., 2019).

Mahbod et al. compared the effect of different preparation methods on the total amount of protein and HGF to introduce the best method for AME preparation. Also, they studied the effect of storage conditions on AME by testing the stability of the HGF under different periods and temperatures (Mahbod et al., 2014). In this study, they first dried some AMs partially (PDAM) and others wholly (CDAM) and then homogenized some of CDAM and pulverized the remaining CDAM in addition to PDAM. According to their results, pulverization of the AM to prepare AME results in 20% more extractable factors in comparison with homogenization as the cellular damage caused by liquid nitrogen results in more protein and HGF emerge. In addition, pulverization is a much easier method, and repeating it up to three times will result in more extractable HGF (almost double). Furthermore, a comparison of different storage conditions revealed that HGF is resistant to repeated freeze-thawing. Additionally, they concluded that while storage temperature does not have any significant effect on HGF level, keeping AME at −170°C results in the least drop in HGF after 6 days. However, this factor is unstable over long-term storage at −170°C. Finally, they showed that the utilization of a 0.2-μm filter for sterilization of the AME has no significant effect on HGF and protein levels. This result indicates that it is possible to prepare AME under unsterile conditions.

### Amniotic Membrane Extract Applications

Here we have reviewed the most significant results of the studies conducted on the therapeutic effect of AME in different fields such as ocular surgery, wound healing, and stem cell expansion. A summary of this information is reported in Table 3.

**TABLE 3:** Summary of studies based on AME.

**Author, year**	**Therapeutic goal**	**Experimental settings**	**Target tissue/cells**	**Conclusion**	**References**
Chang, 2002	Inflammatory skin diseases	*In vitro*	HaCaT cells	AME can be utilized to treat inflammatory skin diseases such as UV-induced skin diseases as it decreases the induction of iNOS mRNA and generation of NO in HaCaT cell by UVB radiation and can protect cells from death or morphological alteration	Chang et al., 2002
Li, 2008	Stem cell preservation and expansion	*In vitro*	AMSCs	AME like AM has the potential to help AMSCs maintain their progenitor status and can reverse differentiated myofibroblasts to a fibroblast phenotype	Li et al., 2008
He, 2008	Anti-inflammatory agent	*In vitro*	RAW 264.7 cells	AME retains anti-inflammatory activities and does so by downregulating activation and inducing apoptosis in macrophages	He et al., 2008
He, 2009	Ocular surface reconstruction	*In vitro*	Human corneal fibroblasts, RAW 264.7 cells	The HC-HA complex is an active component in AM responsible for the suppression of TGF-β1 promoter activity, linkable to its anti-scarring and anti-inflammatory effect	He et al., 2009
Sheha, 2010	Chemical ocular burn	Non-comparative interventional case series	Human eyes	Addition of AME to the standard treatment of mild-to-moderate cases of acute chemical burns results in a reduction of pain, haze, and inflammation and promotes epithelialization	Sheha et al., 2010
Choi, 2013	Wound healing	*In vivo*	Sprague Dawley rats	In comparison with the commercial product, the double-layered AME-loaded wound dressing enhanced wound healing	Choi et al., 2014
Xiao, 2013	Dry eye	*In vivo*	BALB/c mouse	Topical application of AME on BAC-induced dry eye resulted in improved clinical symptoms of dry eye, reduced corneal inflammation, decreased squamous metaplasia, protected corneal epithelial cells and increased their proliferation, and increased the density of goblet cells	Xiao et al., 2013
Kang, 2013	Wound healing	*In vitro* and *in vivo*	Primary human foreskin fibroblasts New Zealand white rabbit	Intradermal injections of AME fluid on wound sites resulted in increased wound closure rate and promoted epidermal and dermal regeneration without causing undesirable hyperproliferation of damaged tissue	Kang et al., 2013
Mahbod, 2014	HGF content of AME	*In vitro*	–	The extraction method of AME and its storing conditions has a direct influence on its extractable components.	Mahbod et al., 2014
Tauzin, 2014	Chronic leg ulcers	*In vitro*	Normal and ulcer fibroblasts	Although AME is beneficial in leg ulcer treatment clinically, in this study, it barely stimulated ulcer fibroblasts	Tauzin et al., 2014
Dudok, 2014	Corneal surface injuries	*In vitro*	Human corneal epithelial and limbal cells	HCE cells healed faster after mechanical injury when they were cultured with AME	Dudok et al., 2015
Lee, 2016	Ocular surface disorders	*In vitro*	Human corneal epithelial cells	Homogenized AME of less than 3 kDa had a higher capacity in the reduction of inflammation	Lee et al., 2016
Vojdani, 2016	Stem cell therapy	*In vitro*	HUCBMSC	AME has the potential to enhance the proliferation capacity of HUCBMSCs without influencing their morphology and differentiation capacity	Vojdani et al., 2016
Go, 2016	osteogenic effects	*In vitro*	MG-63	Unlike CME, the EGF content of AME negatively regulated the osteogenic differentiation of MG-63 cells. However, it can be modified with EGFR inhibitors to modulate the bone density or calcification during bone regeneration	Go et al., 2016
Yadav, 2017	The antibacterial effect of AME against S. pneumonia	*In vitro* and *in vivo*	Microtiter plate assay and OM rat model	AME/CME contains essential antimicrobial proteins and peptides to inhibit S. pneumoniae growth in both planktonic and biofilm states	Yadav et al., 2017
Litwiniuk, 2017	Cell growth	*In vitro*	HaCaT, Wi-38, HECa-10	The placental portion of AM stimulates both fibroblasts and keratinocytes and is best suited for applications related to wound healing. On the other hand, the cervical portion of AM provide from C-section is a better option for the treatment of ocular diseases as it stimulates epithelialization	Brown et al., 1989
Baradaran-rafii, 2017	LSC transplantation	*In vivo*	Human eyes	Application of AM as a supporter (niche/scaffold) and AMEED as the promoter of limbal/epithelial cell growth may be a promising surgical procedure for LSC cultivation	Baradaran-Rafii et al., 2018
Laranjeira, 2018	Allergic disorders	*In vitro*	Human PBMCs	AME induces anti-inflammatory effect on T cells	Laranjeira et al., 2018
Motlagh, 2018	Stem cell therapy	*In vitro*	Decidual MSCs	Coatings based on AME maintain or reduce the size of DMSCs and promote their proliferation, osteogenic, and adipogenic differentiation	Shakouri-Motlagh et al., 2019
Faridvand, 2018	Myocardial hypoxia injury	*In vitro*	H9c2 cardiomyocytes	Proteins present in AME have cardioprotective effects in hypoxic conditions by reducing oxidative stress and inflammatory response and modulating apoptosis	Faridvand et al., 2018
Farzan, 2018	Wound healing	*In vivo*	Rat skin	AME as well as deferoxamine has the potential to induce angiogenesis during wound healing	Farzan et al., 2018
Asl, 2019	Corneal surgery and cell therapy	*Ex vivo* and *in vivo*	LSCs and rabbit	AMEED enhances LSC proliferation and decreases epithelium healing duration by 1 day in comparison to the control group	Asl et al., 2019
Fardivand, 2019	Myocardial hypoxia injury	*In vitro*	H9c2	AME proteins protect cardiomyocytes in hypoxic conditions through the regulation of HO-1 by Nrf2 activation	Faridvand et al., 2019
Fardivand, 2020	Cardiotoxicity	*In vitro*	H9c2	AME has the potential to suppress the cardiotoxicity induced by DOX through inhibition of apoptosis and oxidative stress	Faridvand et al., 2020
Liu, 2020	Dry eye disease	*In vitro*	Human corneal epithelial cells	Through the upregulation of MMP-8 and downregulation of IL-1β and TNF-α, AME protects corneal epithelial cells against benzalkonium chloride	Liu et al., 2020
Park, 2020	OM	*In vitro*	ME mucosa of rats	Possibly AME exerts anti-proliferative and anti-inflammatory effects on infected ME mucosa	Park et al., 2020
Shabani, 2020	Ocular surface disease	*In vitro*	HUVECs	AME loaded chitosan-dextran sulfate nanoparticles decreased the proliferation of endothelial cells	Shabani et al., 2020

#### Ophthalmology

Amniotic membrane is rich in growth factors and structural proteins that influence the corneal healing process from different aspects such as the promotion of re-epithelialization, LSC migration, inhibition of cell apoptosis, and maintenance of epithelial progenitor cells within the LSC niche (Rauz and Saw, 2010; Malhotra and Jain, 2014). Like AM, AME has beneficial bioactive factors efficient in the treatment of ocular surface disorders. As these factors suppress inflammation and neovascularization and promote epithelialization, some studies have shown that AME is a useful substrate for ocular chemical burn treatment (Kim and Tseng, 1995; Choi et al., 2011; Westekemper et al., 2017). According to these studies, AME has a direct influence on decreasing ocular surface inflammation and symptomatic relief. Moreover, its re-epithelialization effect induces proliferation and differentiation in corneal epithelial cells, and it can suppress neovascularization in the cornea after mild to moderate chemical burns (Jiang et al., 2006; Choi et al., 2009; Liang et al., 2009; Dudok et al., 2015). Additionally, it has been observed that AME has protective effects against dry eye disease (Liu et al., 2020).

Although AM transplantation (AMT) is one of the methods for ocular surface treatment in the case of an ocular chemical burn, it may also cause surgical trauma. Besides, the topical use of AME is a much simpler approach as it does not require surgical intervention and has lower morbidity (Mahbod et al., 2014). On the other hand, in comparison with AMT, AME has a comparable effect on epithelialization, suppression of inflammation, and corneal neovascularization (Jiang et al., 2006; Shahriari et al., 2008). AME has also been applied in the form of AME eye drop (AMEED) to treat ocular disorders. Unlike AMT, AMEED makes it possible to deliver therapeutic substances for a more extended period without any surgical intervention (Kordić et al., 2013; Xiao et al., 2013; Dudok et al., 2015). However, AMEED lacks the physical and structural properties of AM (Baradaran-Rafii et al., 2018).

To evaluate AME efficiency in the treatment of acute ocular chemical burn, Sheha et al. (2010) conducted a study. In this study, they added AME to the conventional treatment of acute ocular chemical burn after 2 days of injury and reported that not only did it reduce the pain, but also a reduction in the inflammation was observed in all of the cases. In addition to this, they reported rapid healing of the epithelial in the defect site (within 11 days on average), and no neovascularization in the follow-up period was observed.

Additionally, AME has been applied in some studies with a focus on cornea injury. For instance, it has been noted that AMEED improves the healing of the corneal persistent epithelial defects (Kordić et al., 2013). Dudok et al. (2015) conducted a study to evaluate primary human corneal epithelial (HCE) cell’s response to AME in case of ocular surface injuries and to provide evidence of the safety and cellular benefits of AME on HCE cells. They proved that 0.1% AME solution has a significant influence on mechanical cellular injury due to its effect on epithelialization (Figure 4B). Based on their results, pretreatment of HCE and limbal cell cultures with 0.1% AME prior to tertiary butyl hydroperoxide (t-BOOH) treatment enhances cellular metabolic activity in comparison with cells treated with t-BOOH alone (respectively 73.3% vs. 66.0% and 91.0% vs. 82.0%). In a recent study, Shabani et al. (2020) utilized nanoparticles to release AME in a more controlled manner for the treatment of cornea surface injuries. According to their results, chitosan–dextran nanoparticles containing AME were more effective than AME alone in the inhabitation of corneal neovascularization.

Moreover, due to its anti-inflammatory effect, AME may be a suitable therapeutic option for the treatment of dry eye as inflammation is the primary cause of this disease. This inflammation may be induced by squamous metaplasia, epithelial apoptosis, or goblet cell loss (Kunert et al., 2002; Pflugfelder et al., 2008; Xiao et al., 2013). Although AMT has been applied for the treatment of dry eye, AME may be a better option as it does not have transplantation complications such as suture-related scars and hospitalization. Xiao et al. (2013) evaluated the therapeutic effect of AME on dry eye induced by benzalkonium chloride (BAC). According to their study, AME is capable of reversing the pathological changes associated with dry eye by suppressing the infiltration of inflammatory cells, decreasing global cell apoptosis, alleviating squamous metaplasia, and promoting epithelial cell proliferation. Overall, they concluded that 1.5 and 3 mg AME per day stabilizes tear film, maintains the integrity of epithelium, and alleviates ocular surface inflammation, which all lead to improvement of the clinical manifestation of BAC-induced dry eye in the mouse model (Figure 4C).

Lee et al. (2016) conducted a study to prove the anti-inflammatory effect of AME on human corneal epithelial cells (hCECs) and showed that it was a more efficient anti-inflammatory agent than negative control without inducing apoptosis. Besides, they attempted to identify which part of AME is responsible for this effect. According to their data, the fraction of AME smaller than 3 kDa, which included diverse molecules such as peptides, amino acids, and nucleotides, had more therapeutic, especially anti-inflammatory effect in comparison with larger molecules. Altogether, they proved that AME is a suitable therapeutic approach for mild ocular surface disorders, which are combined with inflammation, such as dry eye syndrome.

Amniotic membrane has been known as a substrate that can support LSC expansion (Plummer, 2009). Although the routine method for LSC expansion involves FBS usage (De Luca et al., 2006; Baylis et al., 2011), it accompanies some challenges such as possible disease transmission and accumulation of bovine antigens, which can lead to activation of the immune response, resulting in transplantation failure (Gregory et al., 2006; Sundin et al., 2007). Thus, a suitable replacement for FBS could be AM or AME, which not only are free from animal antigens but also promote ocular surface reconstruction (Paolin et al., 2016). Additionally, researches indicate that AME reduces inflammation, induces re-epithelialization, and improves patients’ symptoms within 15–20 days after treatment (Liang et al., 2009; Kordić et al., 2013; Xiao et al., 2013). The molecular mechanism of this therapeutic effect of AME has been reviewed by Tseng (Mamede and Botelho, 2015). Asl et al. (2019) evaluated the effect of AMEED on *ex vivo* LSC expansion. According to their results, the optimum dose of AMEED for LSC culture was 0.1 mg/ml, while in an *in vivo* model of rabbit, this dose increased to 1 mg/ml. Additionally, they proved that AMEED limits LSC differentiation, and as AMEED growth factors have a dose-dependent effect, their accumulation should be avoided. Another exciting outcome of their study was that AMEED growth factors are stable for at least 10 months at −70°C, 7 days at 2–8°C, and 2 days at room temperature. Overall, AMEED which increases LSC proliferation *in vitro* and accelerates re-epithelialization *in vivo* is a much more straightforward, more convenient, and less complicated approach in comparison with AMT for corneal defects as it is not associated with progression of corneal surface disorders, corneal thinning or perforation, calcification, and inflammation (Sangwan et al., 2007; Kaup et al., 2008; Asl et al., 2019). In addition, due to AMT lyse after 1–2 weeks, it requires repeated transplantation while AMEED does not have such an issue.

Baradaran-Rafii et al. (2018) hypothesized that utilization of AM in combination with its extract would influence LSC cultivation in a more sensible and inexpensive way. Conventionally, LSCs are expanded *ex vivo* and transplanted with the lowest differentiation to corneal cells. However, it is an expensive and time-consuming procedure that requires special laboratory devices and is not accessible to all patients (Ramaesh and Dhillon, 2003). To overcome these challenges, Barardaran-Rafii et al. developed an alternative single-step procedure that is accessible for all patients without any expensive laboratory facilities. In their proposed surgery, like the conventional method, a small limbal biopsy, which has been harvested from the healthy eye, is transferred to the damaged eye, which is previously covered with a cryopreserved AM. Unlike the conventional method, they added supplemental AMEED postsurgery to promote corneal epithelial healing. According to their results, in those cases where AMEED was not administrated, a persistent epithelial defect was observed. They concluded that autologous limbal tissue in combination with AM as a niche and AME as a supporter could be helpful for less expensive, more rapid, and more straightforward *in vivo* cultivation of LSCs.

#### Wound Healing

AM extract has also been investigated for wound healing applications by some groups. It has been applied as a drug in a double-layered wound dressing containing a layer of PVA (6.7%) and an AME-loaded layer of sodium alginate (0.5%) to improve wound healing characters and gel properties (Choi et al., 2014). It was also utilized solely in other studies. Among them is the research conducted by Kang et al. (2013), evaluating the feasibility of freeze-dried AME as a wound healing substrate. According to their results, AME injection promotes epidermal and dermal regeneration while suppresses their over-proliferation and improves the orientation of dermal collagen bundles in a dose-dependent manner.

Additionally, AME is a valuable source for inflammatory skin diseases such as ultraviolet-induced skin diseases (Chang et al., 2002). Chang et al. (2002) have evaluated the effect of AME on the expression of nitric oxide synthase (NOS) mRNA in HaCaT cells, which is expressed during many inflammatory diseases and is triggered by UV radiation. According to their data, AME, at a specific dose, downregulates the induction of this mRNA upon UV irradiation and protects cells from death or morphological changes.

Angiogenesis, which is one of the most crucial parts of wound healing, is particularly challenging in chronic wounds such as diabetic and venous leg ulcers. Various substrates with angiogenic effects have been studied to accelerate the wound healing rate. Among these substrates are AME and deferoxamine. Farzan et al. (2018) compared the angiogenic effect of these agents separately and in combination with each other. According to their results, AME increases angiogenesis by promoting angiogenic indicators. Recent studies have shown that the angiogenic effect of AME is partially attributed to its chemokine contents and growth factors, which induce endothelialization. In comparison with deferoxamine, which has an excellent capacity for revascularization, there is no significant difference between the number of angiogenic markers of AME and deferoxamine, although their mechanism of action is different. It is noteworthy that the combination of these agents did not surpass the single groups in this study.

#### Heart

It has been previously shown that AME has beneficial effects on mechanical cell injuries and suppression of oxidative stress (Dudok et al., 2015). Fardivand et al. conducted a study to evaluate the molecular effect of AME proteins on suppressing H9c2 cells under hypoxic conditions (Faridvand et al., 2018). They showed that while hypoxia alters cardiomyocytes’ viability, apoptosis, oxidative stress, and inflammation, proteins present in the AME can support cells in hypoxic conditions and decrease their apoptosis. Additionally, AME suppressed hypoxia-induced ROS generation. Overall, the cardioprotective effect of AM is associated with its protein content, which is present in AME and can regulate cell apoptosis and inflammatory responses under ischemic conditions. In another study, they examined the mechanism underlying this protective effect of AME proteins on the same cell line (H9c2 cells) under hypoxic conditions (Faridvand et al., 2019). According to their results, the upregulation of HO-1 and Nrf2 genes in AME treatment results in increased cell survival. In a more recent study, Fardivand et al. evaluated the cardioprotective effect of AME against cardiotoxicity induced by doxorubicin (DOX) (Faridvand et al., 2020). According to this study, the protein content of AME, which has the potential to modulate apoptosis, Ca2+ homeostasis, and inflammation, protects H9c2 cardiomyocytes against the cytotoxicity induced by DOX.

#### Leg Ulcers

Amniotic membrane has been clinically utilized for the treatment of chronic leg ulcers because of the influence it has on epithelialization (Mermet et al., 2007). Tauzin et al. (2014) conducted a preliminary study to evaluate the effect of AME on ulcer fibroblast (UF) in comparison with normal fibroblast (NF). Although their study was limited to the use of single-patient cells, their results show that UF was barely stimulated by AME while NF shows some responses. They developed different hypotheses for this phenomenon, such as the absence of appropriate receptors on UF and impaired signal transduction. They concluded that the beneficial therapeutic effect of AM on leg ulcer may be related to the effect of this substrate on keratinocytes and/or the regulation of inflammation. In a recent study, Alamouti et al. (2019) clinically evaluated the efficiency and safety of AME on diabetic ulcers. According to their results, both small (≤500 mm^2^) and big (≥500 mm^2^) wounds significantly healed after 4 weeks of treatment with AME and their treatments were completed after 6 weeks. Although they concluded that AME has a better effect on smaller wounds and attribute to their wound healing by stimulating keratinocyte migration.

#### Mesenchymal Stromal Cells

Stem cell preservation and expansion have always been a challenge in clinical cell therapy approaches. Different approaches have been developed to increase the proliferation capacity of stem cells and preserve their stemness, such as adding growth factors. For instance, bFGF is an additional factor to increase MSC proliferation capacity; however, it also affects their differentiation potential, which is not favorable in many studies (Sotiropoulou et al., 2006; Nguyen et al., 2015). As a substitute, Vojdani et al. (2016) investigated the effect of AME on the proliferation capacity of human umbilical cord MSCs (HUCBMSCs). According to their results, AME has the potential to enhance the proliferation rate of HUCBMSCs without altering their morphology and differentiation potential.

Moreover, the anti-fibrotic effect of AM has partially arisen from its regulatory effect on growth factors that trigger myofibroblast differentiation (Espana et al., 2004). Li et al. proved that the soluble fraction of AME also possesses this potential. They also demonstrated that as myofibroblasts differentiated from AM stromal cells (AMSCs) cultured in a medium which contains AME, they can revert to a fibroblast phenotype (Li et al., 2008). From this data, they have concluded that AM has soluble factors that can control the differentiation of MSC. This action is accomplished by downregulation of TGF-β, which, together with mechanical stress, has an essential effect on myofibroblast differentiation. These findings may lead to the extraction of specific components from AME or AM stromal extract (ASE) to be used in anti-scaring therapies as well as stem cell preservation and expansion.

As the *ex vivo* environment is significantly different from the MSC niche, preservation of MSC potency in *ex vivo* expansion has become challenging. Shakouri-Motlagh et al. (2019) have proposed that the solubilized form of AM/CM can reproduce the natural environment of MSC in a feasible and reproducible way. AM and CM were solubilized through enzymatic digestion with pepsin, as it does not affect ECM bioactivity. As AM and CM have different compositions, the resulting solutions and environments had different bioactivities. According to their results, AME contains more protein, while CME is rich in GAGs. In addition, coatings produced from a 0.5-mg/ml AME induced the most proliferation in MSC, which was even more significant than the proliferation induced by Matrigel. Coatings based on AME were also able to maintain a much smaller MSC – more potent – and regulate its adipogenic and osteogenic differentiation. They concluded that AME is the most suitable substrate for preserving MSC potency.

#### Osteogenesis

The osteogenic effect of AME and CME is the least investigated characteristic of these extracts. Go et al. (2016, 2017) conducted two studies to explore the ability of AME and CME to promote the osteogenic differentiation of osteoblast-like cells (MG-63). According to their results, although both of these extracts contain osteogenic-related growth factors, CME stimulates osteogenic differentiation more than AME (Go et al., 2017). This phenomenon is due to the presence of EGF in AM and its downregulatory effect on osteogenic differentiation of stem cells. However, modification of AME with EGFR inhibitors results in the modulation of osteogenic efficiency and paves the way for regulating bone density or calcification.

## Hydrogel Based on Amniotic Membrane

**FIGURE 5:**
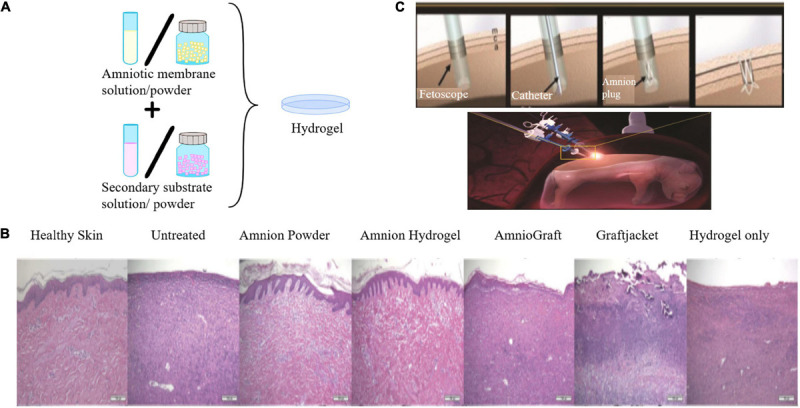
**(A)** Schematic representation of the preparation of AM hydrogel alone or in combination with a secondary substrate. **(B)** Histological images with H&E staining. Comparison of the efficiency of AM powder/hydrogel with commercially available products and other control groups. Skin treated with AM hydrogel and AM powder were very similar to the healthy skin [reproduced with permission from Tseng, 2016. Copyright 2019 Wiley]. **(C)** Placement of the AMED in the injured site of the fetal membrane by minimally invasive fetoscopic surgery [reproduced with permission from Lee et al., 2018. Copyright 2018 Wiley].

In many cases, fresh, freeze-dried, or cryopreserved AM sheets are used for various clinical applications. However, they are associated with some challenges, such as the difficulty of handling without folding or tearing it before placing it on the injury site and its fixation on the injury site for a prolonged period (sutures, glue, or additional bandaging). Aside from AME, recently, a new strategy has been developed to eliminate these challenges by employing hydrogels based on AM. These hydrogels are usually formed by digesting the dAM with pepsin or other methods (based on AME formation) to integrate the benefits of the hydrogel structure with the growth factors and nutrients of the AM (Figure 5A). The produced hydrogel, which can be prepared alone or in combination with carriers for improved biological, mechanical, or gelation properties, has similar properties as collagen and fibrin (Murphy et al., 2017). The summary of the studies based on AM hydrogels is provided in Table 4.

**TABLE 4:** Summary of studies based on AM hydrogels.

**Author, year**	**Therapeutic goal**	**Experimental settings (target tissue/cells)**	**Secondary biomaterial**	**Conclusion**	**References**
Hussin, 2011	Cartilage TE	*In vitro* (primary chondrocytes)	Fibrinogen	The hydrogel-based on AM and fibrin not only secretes cartilage-specific ECM and has significant amounts of GAGs but also preserves cellular phenotype and has a reasonable biodegradation rate	Hussin et al., 2011
Ryzhuka, 2017	Cell delivery and TE	*In vitro* and *in vivo* (PMSCs, Sprague Dawley rat)	–	AM hydrogel supports cellular growth and maintains the normal morphology and physiology of the embedded cells and does not induce any inflammation	Ryzhuk et al., 2018
Murphy, 2017	Wound healing	*In vitro* and *in vivo* (human dermal fibroblast and keratinocyte, mice)	HA	HA-AME hydrogel accelerates wound closure through increasing epithelialization and decreasing contraction and results in smaller average vessel areas	Murphy et al., 2017
Lee, 2018	Fetal membrane healing	*In vivo* (pregnant miniature swine)	PCL framework	AM gel successfully seals the defect site in the fetal membrane and stop AF leakage	Lee et al., 2018
Murphy, 2019	Wound healing	*In vivo* (pig)	HA	HA-AME and AM powder both better stabilize the wound in comparison with other treatments	Tseng, 2016
Toniato, 2019	Articular cartilage TE	*In vitro*	Chitosan	Hybrid hydrogel based on dAM and Ch has the high swelling capacity and elastic modulus	Toniato et al., 2019
Rahman, 2019	Burn wound healing	*In vitro* and *in vivo* (HaCaT, HFF1, Wistar rats)	Aloe vera	Wound dressing based on AM and AV accelerated wound closure with minimum scar formation	Rahman et al., 2019
Henry, 2019	Post-MI tissue repair	*In vitro* and *in vivo* (BAECs, rat)		Injectable and thermoresponsive AM hydrogel improves cardiac contractility and decreases fibrosis	Henry et al., 2020
Rana, 2020	Burn healing	*In vitro* and *in vivo* (RBC, Wistar rat)	Collagen	Hydrogel based on AM and collagen increases the rate of wound healing mainly when it is utilized with a wound dressing membrane	Rana et al., 2020
Lei, 2020	Vascular graft	*In vitro* and *in vivo* (HUVEC, RBC, New Zealand rabbit)	AlgSr/PAM	This graft is resistance to enzymatic degradation and possesses anti-calcification effect, activates platelet and hemolysis, and enhances vascular remodeling and repair	Lei et al., 2020
Peng, 2020	Vascular graft	*In vitro* and *in vivo* (HUVECs, HASMCs, New Zealand rabbits	ADA/REDV	This graft has enhanced mechanical strength and resistance to enzymic degradation. It accelerates endothelialization, and the addition of REDV to this structure stimulates natural anticoagulant substances on naturally derived blood vessels.	Peng et al., 2020

One of the pioneers in this field is Hussin et al. (2011), with their 3D scaffold for cartilage TE applications. This scaffold was composed of AM and fibrin and two biodegradable and biocompatible materials and had a similar structure to hyaline cartilage. The results of this study show that this structure not only secreted cartilage-specific ECM and GAGs but also preserved cellular phenotype and had a suitable biodegradation rate (full, partial degradation at day 30). Another study has shown that AM-based hydrogels could have the required mechanical properties essential for articular cartilage TE. In the study conducted by Toniato et al. (2019), a hybrid hydrogel based on dAM and chitosan (Ch) has been developed with high swelling capacity (ranged between 333 ± 19% and 368 ± 28%) and elastic modulus (Mean of approximately 80 kPa).

AM-based hydrogels have also been applied for wound healing. In one study, Murphy et al. (2017) utilized UV cross-linked HA hydrogel as a carrier for AME to evaluate its efficiency in a full-thickness murine wound model. This easy-to-apply construct conformed the wound shape and depth after UV exposure and had started to degrade by day 7 after application. According to their data, HA-AME hydrogel accelerated wound closure approximately by 3% compared to HA-treated and untreated wounds through increased epithelialization and decreased contraction. Another interesting outcome of their study was that HA-AME-treated wounds had an overall smaller average vessel area in comparison with HA-treated wounds and untreated wounds. Following this study, Murphy et al. conducted another experiment to evaluate the influence of HA-AME over AM powder, AmnioGraft, and HA hydrogel on a full-thickness porcine skin wound model (Tseng, 2016). According to their data, HA-AME and AM powder both better stabilized the wound at days 4 and 7 in comparison with other treatments. Like the former outcomes, HA-AME, and this time AM powder, which were much easier to handle compared to other products, resulted in rapid wound closure rates with the formation of mature epidermis and dermis similar to healthy skin (Figure 5B).

Rahman et al. (2019) have conducted another study based on AM hydrogels for the development of a cost-effective and straightforward wound dressing for burn healing. Their hydrogel is a composition of AM and aloe vera (AV), which has widely been applied for burn healing. Based on their outcomes, this hydrogel had no cytotoxicity, and no edema or erythema was observed after its application for 7 days. According to their *in vivo* study, AM had lower inflammation (even lower than the AM+AV-treated group) and scar formation. On the other hand, the AM+AV-treated group had a similar healing velocity to AM but with a higher epithelialization rate (on day 30). Another wound dressing for burns based on AM has been developed by Rana et al. (2020). This hydrogel was formed by the addition of a solution based on AM and collagen powder to the gelling agent (CMC-Na) and utilized in combination of a chitosan/collagen-blended membrane or alone. According to their data, this biocompatible hydrogel resulted in rapid wound healing in a rat model with complete re-epithelialization and wound contraction.

Recently, AM-based hydrogels have been employed for the treatment of cardiovascular diseases. For instance, Henry et al. (2020) developed an injectable, thermoresponsive AM hydrogel to improve cardiac regeneration after myocardial infarction (MI). Using ultrasound-guided injection, they administrated AM hydrogel into rat MI hearts and evaluated its effect in comparison with PBS. Based on their data, this hydrogel significantly improved cardiac contractility and decreased fibrosis (*p* < 0.05). In another study, a dAM hydrogel modified with polyacrylamide-alginate (AlgSr/PAM) was utilized as a vascular graft in a rabbit model (Lei et al., 2020). This thermosensitive construct had high mechanical strength and bioactivity in addition to low swelling ratio. According to Lei et al., this graft showed resistance to enzymatic degradation and possessed an anti-calcification effect. Moreover, while it inhibited platelet activation and hemolysis, this graft enhanced the adhesion and expansion of endothelial cells as well as vascular remodeling and repair. In a similar experiment, Peng et al. (2020) used D-double-cross-linked decellularized AM hydrogel grafted with REDV(ArgGlu-Asp-Val) polypeptides which showed excellent mechanical strength and resistance to enzymatic degradation and inhibited hemolysis and coagulation (similar characteristics to the vascular graft developed by Lei et al.). Also, the addition of REDV to this structure stimulated natural anticoagulant substances on naturally derived blood vessels.

Amniotic membrane based hydrogel has also been utilized as a vehicle for cell delivery (Ryzhuk et al., 2018). In this experiment, Ryzhuka et al. developed an AM-ECM hydrogel via pepsin digestion of dAM and compared its effect to conventional collagen and fibrin hydrogel matrices. Based on their data, AM hydrogel gelation time is longer than fibrin gel and shorter than collagen. Their *in vitro* assessment shows the ability of AM-based hydrogel to support the cellular growth and high rates of cellular propagation. According to their results, AM-ECM is a biocompatible hydrogel that supports the natural morphology and physiology of a variety of stem cells. Besides, no tissue inflammation and immune cell activation were observed in their *in vivo* assessment on the Sprague Dawley rat model.

Another interesting application of AM hydrogel is actually to restore AM defects during pregnancy (Lee et al., 2018). This iatrogenic preterm premature rupture of the fetal membranes results in premature births and requires external intervention to stop amniotic fluid (AF) leakage. Lee et al. have developed a 3D-printed construct, which is called biocompatible amnion-analogous medical device (AMED), consisting of a PCL framework and dAM hydrogel to seal the defect site and preserve fetal survival (Figure 5C). To do this, dAM gel was injected in the AMED with a syringe and became cross-linked (after 30 min at 37°C) and ready for application at the injury site. According to their results, it was possible to deliver this device through a fetoscope-sized catheter in 33.62 ± 11.36 s (10.26 times faster than amniotic graft transplantation). Following that, this gel successfully sealed the defect site in all animals (pregnant miniature swine), and they recovered entirely after surgery and maintained a healthy condition until the end of the pregnancy.

## Discussion

The amniotic membrane contains many proteins and growth factors and, as a result, possesses interesting properties and structure. This low immunogen and biologically viable structure is easily accessible and has a reasonable price. These characteristics have made AM a popular choice for medical purposes. Its application in ophthalmology and skin care has a long history. Due to its desirable results, nowadays, there are commercially available AM products for clinical use. Additionally, although it has relatively poor mechanical properties, its utilization for bone and cartilage regeneration and internal surgeries is developing as a result of recent modifications to its structure.

With growing interest in AM applications in TE and regenerative medicine, some modifications have been made to improve its properties with the addition of other biological or synthetic materials. In this review, we have categorized and described these modifications and their final effect on the AM structure. Based on our research, these modifications can be categorized as composites based on AM, AME, and hydrogels based on AM. Composites based on AM are consist of an amniotic layer, which is (1) modified by a coating of particles or electrospun or casted layers, (2) applied as a coating over another structure, or (3) applied as particles to enhance the biological properties of another material. For instance, the AM antibacterial effect has been improved by the addition of silver nanoparticles and Calvanin A, its adhesion capacity has been regulated by insertion of halofuginone (alone or with chitosan), and its biocompatibility has been specialized by addition of tissue-specific ECM. Additionally, its wound healing features have been improved by the insertion of silk. Moreover, its mechanical properties and degeneration rate have been improved by adding a polymeric layer (such as electrospun PCL, PLCL) or gels (GelMA). On the other hand, AM can improve the inflammatory response, adhesion capacity, blood compatibility, and biocompatibility of other materials when it covers them or mixes with them to form a scaffold.

Additionally, AME, which is much easier to use and has the same effects of intact AM, can be formed into a hydrogel, which is easier to handle, store, and sterilize. AME has been especially useful in ophthalmology as it does not have the complications of surgeries, yet its best effect is when it is utilized in combination with AMT. In addition to the anti-inflammatory, antibacterial, anti-scarring, wound healing, and cardioprotective effects of AME, it is suitable for the regulation of stem cell differentiation and expansion. Hydrogels based on AM are mainly provided from the AME and, like AM and AME, posses interesting characteristics suitable for cartilage TE, vascular TE, cell delivery, cardiac tissue regeneration, and wound healing. It has even been applied as a structure to heal and seal fetal membrane injuries.

Based on our findings, although intact AM has always been a suitable option for many clinical and experimental studies, but by addition of extra modifications, it is possible to widen AM applications in the fields such as bone repair, cartilage TE, urinary bladder TE, oral defects, and many other areas that have been mentioned in this review. The growing number of studies that have applied AM with a modified structure highlights the need for further investigation of these structures and their importance. In this review, we have summarized and discussed these modifications.

## Author Contributions

All authors listed have made a substantial, direct and intellectual contribution to the work, and approved it for publication.

## Conflict of Interest

The authors declare that the research was conducted in the absence of any commercial or financial relationships that could be construed as a potential conflict of interest.

## Abbreviations

ADA: alginate dialdehyde
ADA/REDV-AM: ADSCs: human adipose tissue-derived mesenchymal stromal cells
AM: amniotic membrane
AME: AM extract
AMEED: AME eye drop
AMSCs: amniotic membrane mesenchymal stromal cells
AMT: AM transplantation
ASA: acetylsalicylic acid
ASE: AM stromal extract
AT-MSCs: adipose tissue-derived mesenchymal stromal cells
AV: aloe vera
AlgSr/PAM: polyacrylamide-alginate
BAC: benzalkonium chloride
BAECs: bovine aortic endothelial cells
BAMCSM: bovine AM-chitosan membrane
bFGF: basic fibroblast growth factor
CAM: chick chorioallantoic membrane
CBMSCs: cord blood-derived mesenchymal stromal cells
C/CS: collagen-chondroitin sulfate
CDAM: completely dried AM
CG: collagen-glycosaminoglycan
CM: chorion membrane
CME: CM extract
CS: chondroitin sulfate
dAM: decellularized amniotic membrane
dBAM: decellularized bovine AM
dAP: decellularized amniotic particles
DOX: doxorubicin
EDC/NHS: *N*-(3-dimethylaminopropyl)-*N*-ethylcarbodiimide/*N*-hydroxysuccinimide
EDTA: ethylenediaminetetraacetic acid
EGF: endothelial growth factor
EPDC: human epicardial derived cells
FBS: fetal bovine serum
FG: fibrin glue
GelMA: methacrylated gelatin
HA: hyaluronic acid
HaCaT: human keratinocyte cell line
HASMC: human aortic smooth muscle cell
hcECM: human cardiac ECM
hCE: human corneal epithelial
hCECs: human corneal epithelial cells
hCF: human cardiac fibroblasts
HEK: human embryonic kidney 293 cell
HFF1: human fibroblast cell line
HGF: hepatocyte growth factor
HL1: murine cardiomyocyte-like cells
HUCBMSCs: human umbilical cord mesenchymal stromal cells
HUVEC: human umbilical vein endothelial cell
IL: interleukin
KGF: keratinocyte growth factor
LSC: limbal stem cell
ME: middle ear
MenSCs: menstrual blood-derived stem cells
MIF: migration inhibitory factor
mRNA: messenger RNA
NTHi: non-typeable Haemophilus influenza
NF: normal fibroblast
NOS: nitric oxide synthase
OM: otitis media
PAAc: poly(acrylic acid)
pAM: particulated AM
pASC: porcine adipose-derived stem cells
PBMCs: peripheral blood mononuclear cells
PCL: poly( ε-caprolactone )
PDAM: partially dried AM
PDGF: platelet-derived growth factor
PEG: polyethylene glycol
PEGDGE: poly(ethylene glycol) diglycidyl ether
PGE2: prostaglandin E2
PLA: poly(lactic acid)
PLLA: poly(L-lactic acid)
PLGA: poly(D,L-lactide-co-glycolide)
PLGC: poly-(lactide-co-glycolide-co-caprolactone)
PLCL: poly(L-lactide-co- ε-caprolactone )
PMSCs: placenta-derived mesenchymal stromal cells
PP: polypropylene
PSIS: porcine small intestinal submucosa
PVA: polyvinyl alcohol
RBC: red blood cell
REDV: arginine–glutamic acid–aspartic acid–valine
ROS: reactive oxygen species
SDS: sodium dodecyl sulfate
SMC: smooth muscle cell
SS: stainless steel
t-BOOH: tertiary butyl hydroperoxide
TE: tissue engineering
TGF-β: transforming growth factor beta
TIMP: tissue inhibitors of metalloproteinases
TNF-α: tumor necrosis factor alpha
UF: ulcer fibroblast
UV: ultraviolet
VEGF: vascular endothelial growth factor.
